# Measuring sustainability in healthcare: an analysis of two systems providing insoles to patients with diabetes

**DOI:** 10.1007/s10668-020-00901-z

**Published:** 2020-08-25

**Authors:** Stefan Hellstrand, L. Sundberg, J. Karlsson, R. Zügner, R. Tranberg, Ulla Hellstrand Tang

**Affiliations:** 1 Nolby Ekostrategi, Tolita 8, 665 92 Kil, Sweden; 2 Gothenburg Diabetes Association, Mellangatan 1, 413 01 Göteborg, Sweden; 3 The Department of Orthopaedics, Institute of Clinical Sciences, Sahlgrenska Academy, University of Gothenburg, Göteborgsvägen 31, 43180 Mölndal, Sweden; 4 The Department of Prosthetics and Orthotics, Sahlgrenska University Hospital, Falkenbergsgatan 3, 412 85 Göteborg, Sweden

**Keywords:** Healthcare, Sustainability, Diabetes, Diabetic foot, Non-communicable diseases, Sustainable Development Goals

## Abstract

There is an increasing demand to quantify the footprints, ecological, economic and social, in terms of the effect of different interventions in healthcare. The aim of this study was to compare two systems providing patients with diabetes with insoles in terms of their ecological, economic and social footprints. Prefabricated insoles (PRI) were compared with custom-made insoles (CMI). Using a welfare-economic monetary approach, costs were estimated for (1) treatment, (2) travelling to and from the hospital in terms of both fuel and time consumed by the patients and (3) society through emissions contributing to climate change. The proportion of patients/year that could be supplied within the same budget, for each individual treatment, was calculated. The cost of the insoles was 825 SEK (PRI) and 1450 SEK (CMI), respectively. The cost, mean value/patient due to the consumption of patients’ time at the department, was 754 SEK (PRI) and 1508 SEK (CMI), respectively. Emissions, in terms of CO_2_ equivalent, were 13.7 (PRI) and 27.4 (CMI), respectively. Using PRI, a total of 928 patients could be provided/year compared with 500 patients if CMI are used. By using PRI, the cost/treatment was reduced by 46%. The cost of treatment dominated and the cost of time consumed by patients were also substantial. The societal cost of contributing to climate change was of low importance. By using PRI, the needs of 86% more patients could be met within the same budget. Using these methods, the contribution of healthcare systems to the 17 Sustainable Development Goals approved by the UN can be quantified.

## Introduction

Insoles and appropriate footwear are frequently used to prevent pressure-induced diabetic foot ulcers (DFU) and footwear enables people to walk (World Health Organisation 2016). The ability to be physically active is an opportunity for people with diabetes to prevent the further progression of lifestyle diseases and, subsequently, to prevent DFU and by extension amputations. A person with diabetes runs a lifetime risk of developing DFUs of 19–34% (Armstrong et al. 2017). The reported prevalence of DFU varies (1–11%) and is dependent on where in the world, the data are located (International Diabetes Federation 2017a). Promising results show that DFU and amputation can be halved by using a programme that includes appropriate footwear, podiatry, information, early detection and interventions for those at high risk of developing DFUs (Moxey et al. 2011; Bus and van Netten 2016a).

To ensure sustainable development, all services, including healthcare, should be delivered within the planetary boundaries and methods and examples of how to calculate the sustainability effects of different types of intervention that are needed (Whitmee et al. 2015; Hellstrand and Hellstrand Tang 2019). An approach for measuring sustainability performance in healthcare has previously been presented (Hellstrand and Hellstrand Tang 2019). They presented three pathways by which healthcare relates to ecological sustainability.

1. The use of natural resources, such as energy and associated emissions associated with healthcare systems and transportation to and from the healthcare provider, relates healthcare systems to stocks of natural capital and life-support systems.
2. Improved efficiency in healthcare eventually implies less pressure on natural resources for delivering the same healthcare.
3. The human economy through emissions impairs the environment and human health.

The approach is based on a general toolbox for sustainable development, first presented by Hellstrand (Hellstrand 2015). By using the instruments in the toolbox, the performance of any system in relation to national Swedish Environmental Quality Objectives (Miljöportalen 2016), as well as a number of the 17 Sustainable Development Goals (SDG) of the United Nations (UN), can be quantified.

In healthcare, non-communicable diseases (NCD) are a group of diseases with a substantial impact on the health level of societies. These diseases are the cause of 41 million people deaths every year (World Health Organisation 2018). Cardiovascular diseases account for 17.9 million people annually, cancer 9.0 million, respiratory diseases 3.9 million and diabetes 1.6 million. The social and economic costs of diabetes to the individual and to society are significant. The cost per DFU has been estimated at USD 19,000 (1990 price level), according to Apelqvist et al. (1995) and 7147 euros (2005 price level), (Prompers et al. 2008). Lowering the prevalence of diabetes improves social and human capital (Hellstrand and Hellstrand Tang 2019). The prevention of diabetes supports a number of the 17 SDGs of the UN (United Nations 2015).

In the present study, an approach for analysing sustainability performance in healthcare on NCD was applied (Hellstrand and Hellstrand Tang 2019). More specifically, this is made in relation to the prevention of DFUs. In 2019, the global prevalence of diabetes was 463 million people, a number that is expected to increase to 700 million in 2045 (International Diabetes Federation 2019). The increase is dramatic and some contributory factors are lifestyle changes and environmental issues. Endocrine disruptors in food, cosmetics, toys and products have been linked to metabolic disorders such as diabetes (Le Magueresse-Battistoni et al. 2017; European Parliament 2019). Diabetes is related to severe complications such as neuropathy, foot ulcers and amputation (Zhang et al. 2020) and was estimated to affect 131 million people in 2016, 1.8% of the global population (Zhang et al. 2020).

The presence of peripheral neuropathy ranges from 16 to 87% with symptoms such as sensory symptoms, pain and tingling sensations in the feet (International Diabetes Federation 2019). The ability to feel pain helps the individual to protect the feet from sharp objects that could harm the foot. The lack of ability to feel pain, in combination with reduced blood circulation, increases the risk of developing foot ulcers (International Working Group on the Diabetic Foot 2019). In 2017, the total number of patients at risk of developing DFUs was approximately 200 million globally (International Diabetes Federation 2017b) and 200,000 in Sweden (Hellstrand and Hellstrand Tang 2019; Hellstrand Tang 2017), based on the assumption that 50% of the patients have peripheral neuropathy. The intervention, the analysed prescription of insoles and appropriate shoes, is recommended in guidelines for patients at risk of developing DFUs (Formosa et al. 2019; International Working Group on the Diabetic Foot 2019). The purpose of the supply is to prevent pressure-induced DFUs (Van Netten et al. 2016).

In the light of pandemics, such as Covid-19, travelling and social interaction should be minimised for risk groups in the supply of insoles as well, thereby enabling patients to follow official recommendations (Rogers et al. 2020).

The aim of this study was to compare two systems providing patients with diabetes with insoles in terms of their ecological, economic and social footprints. We present a case that is easy to follow by using numbers that are easy to use, while still being realistic.

## Method and study setting

Sahlgrenska University Hospital provides insoles to patients with diabetes in Gothenburg and the surrounding region. The case is based on costs collected from the department of prosthetics and orthotics (DPO) in 2011. The number of patients in need of this specific healthcare once a year is approximately 10,000, while the number of persons receiving the treatment is estimated at 500. Real-world figures relating to the number of patients at risk are not registered. The estimate of 10,000 is related to the number of people with diabetes in Gothenburg, 20,000 (Hellstrand Tang 2017), of whom 50% have neuropathy.

This model is based on the cost per DFU treated of USD 19,000 (1990 price level) according to Apelqvist et al. (1995) and 7147 euros (2005 price level), (Prompers et al. 2008). For two reasons, we chose the estimate from Apelqvist et al. when we discuss the benefits of delivering appropriate insoles to patients with diabetes. The first is that data emanate from the Swedish system, which is a subsystem of the Swedish system we study. The second is that it is a more thorough analysis than most, including treatment costs from when the first DFU is treated and for the following 3 years. The value, 19,000 US$, is an average per DFU for the total population of patients with DFUs in the study by Apelqvist et al. In 1990, 19,000 US$ was worth around 110,000 SEK.^1^

It is assumed that an appropriate supply of insoles, as a part of an effective prevention strategy, will prevent the development of DFUs in 5% of the population studied (Armstrong et al. 2017). In other words, if the total population in need is 10,000, as is the case in the Gothenburg area, an appropriate supply of insoles will reduce the number of DFUs by 500 per year, all else being equal.

**Fig. 1:**
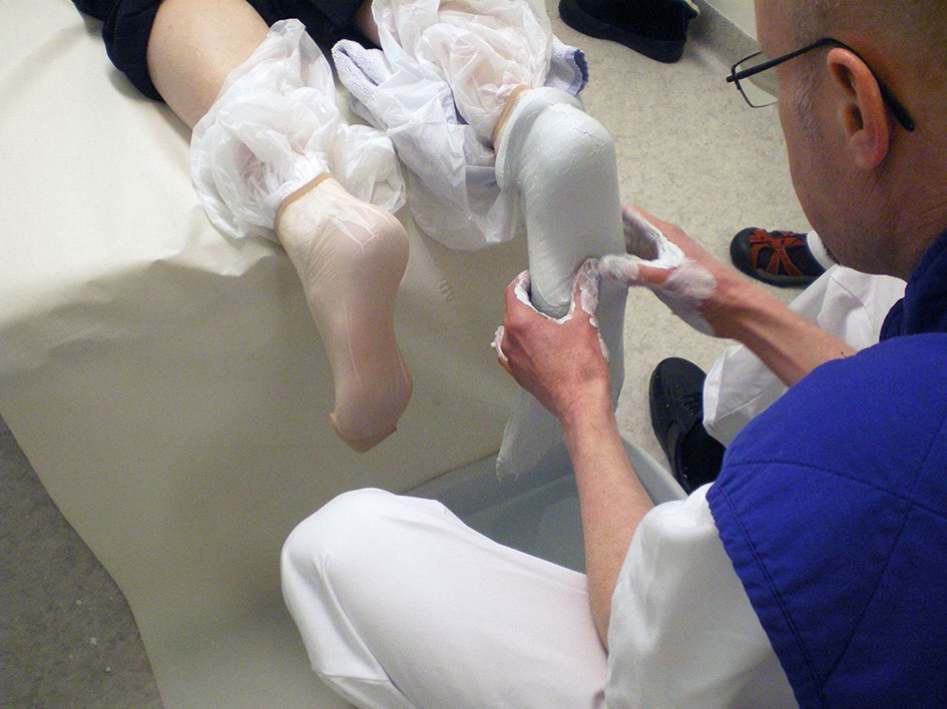
Custom-made insoles were produced on the basis of an individual positive cast (Hellstrand Tang 2017)

**Fig. 2:**
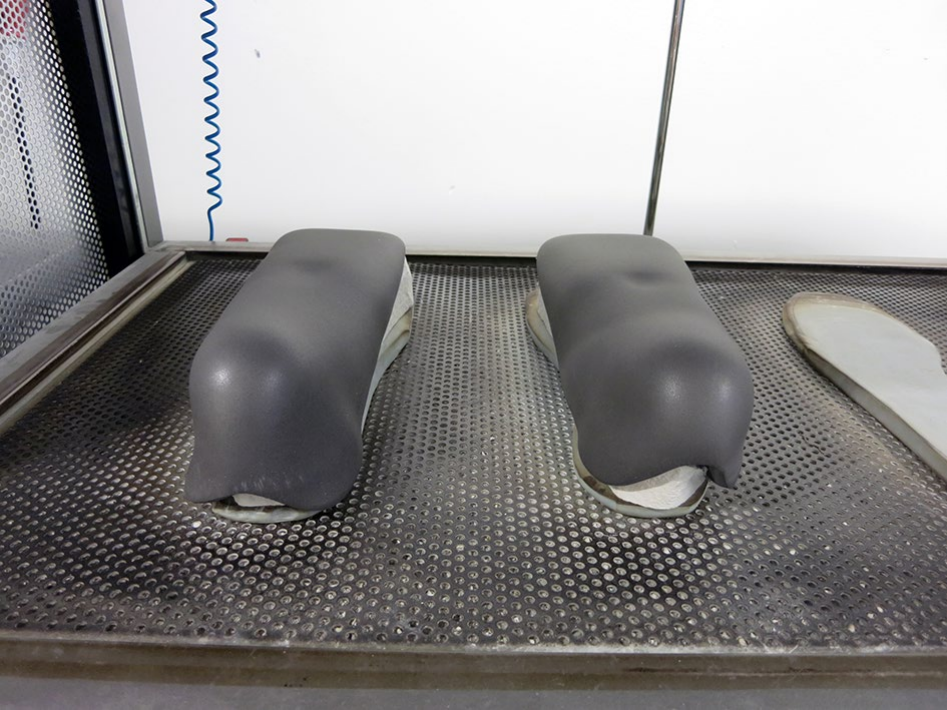
The custom-made insoles were produced in a traditional manner using vacuum heating and grinding (Hellstrand Tang 2017)

In the present example, two systems for meeting the patients’ need for insoles were compared. In the traditional way, patients visit the DPO, the certified prosthetist and orthotist (CPO) makes the assessments and measurements are taken. The custom-made insoles (CMI) are manufactured and fitted in an appropriate shoe on the second visit (Figs. 1, 2) (Hellstrand Tang et al. 2014).

**Fig. 3:**
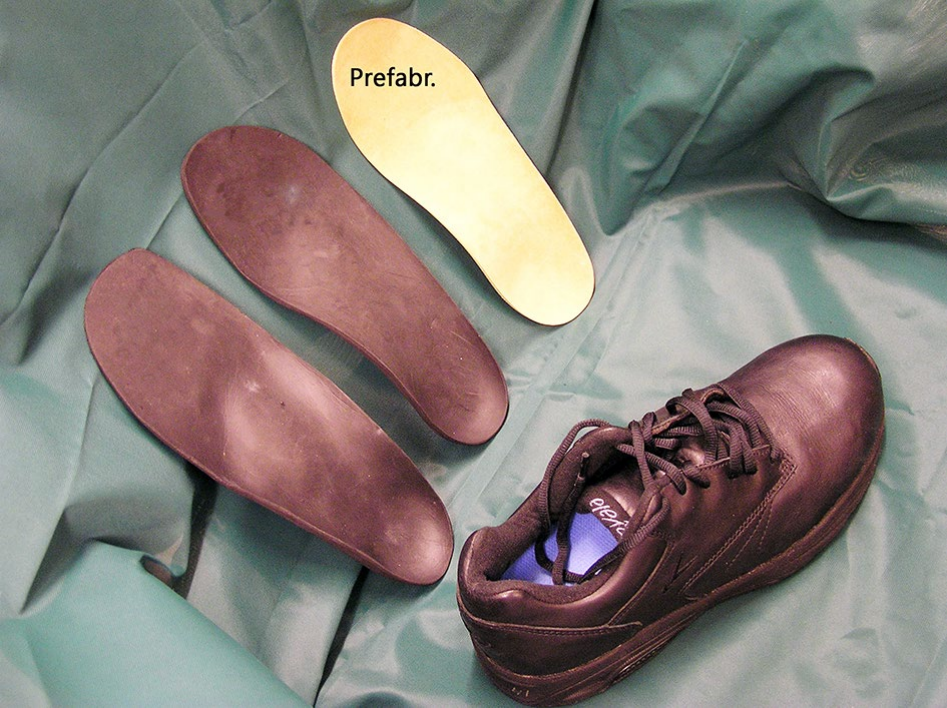
Custom-made (black) or prefabricated insoles were adjusted to fit well in shoes (Hellstrand Tang 2017)

In a new system, prefabricated insoles (PRI) are already delivered on the first visit, Fig. 3. The function, reducing the pressure under the sole of the foot, can be achieved with CMI or PRI (Hellstrand Tang et al. 2014; Van Netten et al. 2020; Paton et al. 2012). The advantages of CMI are that they are (1) individually matched to the form of the foot and have a pressure-reducing effect under the heel (Hellstrand Tang et al. 2014), (2) some patients prefer walking on thicker, cushioning insoles (Gerrard et al. 2020) and (3) adjustment, e.g. medial or lateral support of the foot, is easier with thicker insoles. The disadvantages are that CMI are thicker and as a result require that the shoe comes with removable insoles with a thickness of > 3–5 mm. Furthermore, two visits are required, requiring (1) twice the work of transportation and (2) consuming twice as much of the CPO’s time. Hellstrand Tang et al. (2014) reported that both systems had good pressure-distributing effects under the forefoot and the midfoot and that patients were satisfied with the insoles and used them frequently, independent of whether they were CMI or PRI (Hellstrand Tang et al. 2014).

## Results

Table 1 presents information on the economic aspects of the two alternatives for the provision of insoles in healthcare. Data as they were in 2011, including price level.

**Table 1:** Economic effects for the healthcare provider of two alternatives for delivering one pair of insoles

	Custom-made	Prefabricated
Price per pair of insoles, SEK	1450	825
Labour, hours	2	1
Cost per hour, SEK	570	570
Labour, SEK	1140	570
Total costs, SEK	2590	1395
Number of treatments	500	
Total cost to the healthcare provider, SEK all patients 1 year	1,295,000	
Budget restriction for treatments, no of treatments per year, if same budget in the two alternatives	500	928
Patients needing treatment	10,000	
Percentage of patients whose needs are fulfilled	0.05	0.09

The price per pair of insoles is, for PRI, the price paid, while, for CMI, it is the cost of producing them, including labour.

Labour is the time used when meeting the patients.

By using PRI, the number of patients that are treated within the same budget increases by 86%. With the given budget restrictions, the percentage of patients in need of the treatment whose demands can be met increases from 5 to 9%. Table 2 shows the use of some important ecological resources.

**Table 2:** Some ecological resources consumed by two alternatives for delivering one pair of insoles

	Custom-made	Prefabricated
*Visits per treatment*	2	1
Ecological resources consumed		
*For the provider*		
*Electricity*, kWh per m^2^	130	130
In total for 29 m^2^ locations used, kWh	3770	3770
Per treatment, kWh	6.3	3.15
Per treatment, SEK	6.45	3.22
*District heating*, kWh per m^2^	170	170
In total for 29 m^2^ locations used, kWh	4930	4930
Per treatment, kWh	8.2	4.1
*For the patient*		
Average distance, travel to and from the healthcare provider 60 km		
Total distance per treatment, km	120	60
Petrol/fuel, dm^3^ per 10 km	0.85	0.85
Petrol/fuel, dm^3^ per treatment	10.2	5.1
Petrol/fuel, kWh per treatment	92	46
Petrol/fuel, cost per journey, SEK (price 13.9 SEK per dm^3^)	142	71

Table 3 shows the emission of carbon dioxide for two systems delivering one pair of insoles.

**Table 3:** Emission of carbon dioxide for two systems delivering one pair of insoles in physical terms (kg CO_2_ equivalent) and societal (SEK)

	Custom-made	Prefabricated
*Assimilative capacity*		
Electricity, kg CO_2_ equivalent	0.18	0.09
Petrol/fuel, kg CO_2_ equivalent	27.2	13.6
Total, kg CO_2_ equivalence	27.4	13.7
Societal cost, climate change impact, SEK	41.0	20.5

The cost of 1 kg of carbon dioxide is SEK 1.50, 2006 price level

SIKA. 2009. Värden och metoder för transportsektorns samhällsekonomiska analyser – ASEK 4 SIKA. Rapport 2009:3

The societal cost of the emissions of climate change gases follows the route in previous studies (Hellstrand 2013, 2015). It is based on the preferences expressed by Swedish society to avoid climate change, i.e. costs associated with different policy measures targeting climate change. The total emissions of carbon dioxide equivalents in the system with PRI were estimated at 12,700 kg and, in the system with CMI, 13,700 kg.^2^

In Table 4, the cost to the patient due to the consumption of her/his time associated with the treatment is presented.

**Table 4:** The cost of the consumption of the patient’s time in two systems delivering one pair of insoles

	Custom-made	Prefabricated
Time patient, hours	8	4
*On average*		
Half the time is labour time, hours	4	2
Half the time is leisure time, hours	4	2
SEK per hour labour	275	275
SEK per hour leisure	102	102
*Average cost per pair of insoles, SEK*		
Labour time	1100	550
Leisure time	408	204
Total	1508	754

The estimates are based on SIKA (2009), 2006 price level

SIKA. 2009. Värden och metoder för transportsektorns samhällsekonomiska analyser—ASEK 4 SIKA. Rapport 2009:3

With a new system for treatment that halves the number of visits (i.e. the intervention with PRI compared with CMI), the cost to the patient of the time consumed is halved (Table 4).

Table 5 gives some of the societal costs of the two systems. In Tables 1, 2, 3 and 4, the results are per pair of insoles, i.e. per patient. We assume that, within the same budget, 500 patients per year will receive one pair of insoles with the CMI system and 900 with the PRI system. Table 5 shows the system performance with 500 patients receiving CMI and 900 receiving PRI, respectively.

**Table 5:** Costs in SEK million for two systems delivering insoles to patients with diabetes at Sahlgrenska University Hospital in 2011

	Custom-made		Prefabricated	
	Million SEK	Share of total cost	Million SEK	Share of total cost
Patients treated (no)	500		900	
Cost of delivering insoles, (1295 SEK, from Table 1)	1.300	0.61	1.300	0.63
Cost of petrol/fuel, for travelling to and from the hospital	0.071	0.03	0.064	0.03
Societal cost of contribution to climate change	0.020	0.01	0.018	0.01
Cost of the consumption of the patient’s time	0.75	0.35	0.68	0.33
Total costs	2.141		2.062	
Treatment costs saved for DFUs that are prevented	2.8		5.0	

In the method and study setting, it is assumed that 10,000 patients were at high risk in the Gothenburg area and 5% of these would develop DFUs, 500 per year. The future treatment cost saved for every DFU prevented is 110,000 SEK (estimated from Apelqvist et al. 1995). From this, it follows that

1. with 500 patients treated with CMI, the healthcare system will save 2.8 million SEK in costs avoided due to 25 fewer cases of DFU
2. with 900 patients treated with PRI, the healthcare system will save 5.0 million SEK in costs avoided, due to 45 fewer cases of DFU
3. with 10,000 patients treated, the total need in the Gothenburg area, the healthcare system will save 55 million SEK in costs avoided, due to 500 fewer cases of DFU

In (1) and (2), the cost of delivering this treatment is 1.3 million SEK. The net savings in (1) are therefore 1.5 million SEK, while in (2) they are 3.7 million SEK. If PRI are used, the cost of meeting the needs of 10,000 people is 14.4 million SEK, making the net savings around 40 million SEK.

## Discussion

This study applies a general toolbox supporting the implementation of policies effectively supporting sustainable development. The instruments in this toolbox are generated by the integration of contributions from agricultural sciences, systems ecology, welfare economics, lifecycle assessments, integrative assessments and applied environmental sciences (Hellstrand 2015). Here, these instruments are adapted to evaluate the contribution to sustainable development in healthcare systems. More specifically, they are used to evaluate the ecological, economic and social outcomes within the framework of Agenda 2030 and its 17 SDGs of two ways of meeting the need for the early treatment of patients with diabetes, preventing the development of DFUs. The SDG was approved by the UN in 2015, and it sets goals for local and global development to 2030.

The case reflects a situation from healthcare in Sweden in 2011, more precisely at a DPO. A large part of the service is directed at patients with diabetes at risk of developing DFUs. The recommended treatment includes, in addition to the supply of insoles, appropriate shoes, podiatry and regular controls. In the presence of active foot ulcers, the patients are referred to a multidisciplinary team (Socialstyrelsen 2015; Sveriges Kommuner och Regioner 2018; International Working Group on the Diabetic Foot 2019).

The traditional and common way of addressing the need for insoles is to provide patients with CMI. A comparison is made with a system providing patients with PRI. Both systems reduce high plantar pressure, thereby reducing the risk of developing pressure-induced foot ulcers and the risk of amputation (Hellstrand Tang et al. 2014; Paton et al. 2012).

For the hospital, with a new system (PRI) and with the same budget, the need for insoles of approximately 900 patients annually in Gothenburg can be met, compared with 500 for CMI. The total number of patients in this area needing this treatment is 10,000.

Assuming that 5% of the patients would otherwise have developed DFUs, the increase in the capacity to treat 400 patients reduces the number of DFUs by 20 a year (International Diabetes Federation 2017a; Cavanagh et al. 2005; Singh et al. 2005).

Following the result in Table 5, the “profit” for the healthcare system is 1.5 million SEK for CMI and 3.7 million SEK for PRI. If PRI are chosen, the net result of providing 10,000 patients in Gothenburg with PRI would be a saving of around 40 million SEK.

In an extended analysis, the impact on the quality of life and the impact on the productivity in society of a better health status for 10,000 individuals in need of this treatment in a total population of 500,000 people should be included.

The main difference between the two systems is that the system with CMI requires two visits to the hospital, while PRI only requires one. This affects the use of district heating for the building and electricity for equipment. Energy consumption produces emissions which affect a number of the 16 environmental quality objectives in Sweden (Miljöportalen 2016). It also affects a number of the 17 global SDGs (United Nations 2015) approved by the UN in 2015, which relate to the environment. The UNEP, in collaboration with the WHO, estimates that, in 2012, an estimated 12.6 million deaths, or 23% of the total, were attributable to deteriorating environmental conditions (UNEP 2016). Air pollution dominates.

With a 50% reduction in visits per treatment, the energy costs and associated emissions for each completed treatment are halved. In our example, related to the system for CMI, the energy consumption per treatment expressed in electricity was 6.3 kWh, for district heating 8.2 kWh and for travelling to and from the hospital 92 kWh. So, of the total consumption of energy, 86% related to transportation. The percentages were the same for PRI. With a reduction in visits of 50% per treatment, the use of district heating and of electricity was reduced to 50% as well. It therefore follows that the environmental impact of the total energy use per treatment was dominated by the emissions related to the energy used for transportation to and from the hospital.

Given the real costs and preferences expressed in Sweden, the system with PRI has a total cost of delivering insoles to 900 patients of SEK 2.062 million. Of this, 63% relates to the cost to the hospital delivering the insoles and 33% is the cost to the patient, where the largest part is the cost of the consumption of her/his time. The societal cost of the contribution to climate impact was, however, only < 1% of the total costs. The remaining cost, 3%, was for petrol/fuel, for travelling to and from the hospital. In the system with CMI, the total cost of delivering 500 pairs of insoles was estimated at SEK 2.141 million, where 61% represented costs to the hospital and 35% costs to the patients.

The results in Table 5 suggest that when developing economically efficient healthcare systems, the costs associated with the time consumed by the patient should be considered. In welfare-economic terms, the cost of the contribution to climate change is of minor importance, given the route of calculation behind the results in Table 5.

In both systems, the cost savings to the hospital of delivering insoles to patients with diabetes through future reduced costs of treating DFUs are substantially higher than the cost of delivering insoles. With the low percentage of the need for insoles in the total group of patients with diabetes that is met, the potential for net savings in the healthcare system is substantial by fulfilling the total need for insoles among patients with diabetes. Our assumption is that both systems of delivering insoles will halve the number of DFUs. Different studies suggest that a reduction of this kind is possible. We have allocated this total potential for improvement to the supply of insoles. Clearly, this is too optimistic. Our argument is the need to keep the analysis as simple as possible, in order to make the principal logic easier to understand. However, Bus and van Netten (2016a) suggest that effective preventive measures including the education of patients can reduce the proportion of DFUs by 75% (Bus and van Netten 2016a). If so, our assumption of the impact of delivering insoles as a part of effective prevention on reducing DFUs by 50% is not so unrealistic: With insoles, the risk of DFUs decreases and the patient will also benefit from the opportunity for a higher level of physical activity.

According to the data presented in Table 5, it is suggested that in the system with CMI, the delivery of the service to the 10,000 patients requiring the service would result in annual net savings to the hospital of around 30 million SEK. If, instead, the PRI were used, the net savings would be around 40 million SEK.

If we want to scale up the results to national level, we can simply multiply the results by a factor of 20, as the Swedish population is 20 times larger than the population in the Gothenburg region.

If so, and assuming proportional relations, the national annual net savings in the healthcare system would range from 600 million SEK with the CMI system to 800 million SEK with the PRI system. Note that, this presupposes that as a national average, 5% of the population of patients in need of this treatment receives it, just as in Gothenburg.

The total emissions of CO_2_ equivalents in the system with PRI were estimated at 12,700 kg and, in the system with CMI, 13,700 kg (Table 3).

On average, through photosynthesis, Swedish forests assimilate 169 million tonnes of CO_2_ every year (Hellstrand 2015). With 23 million hectares (ha) of productive forest land, this corresponds to 7350 kg of CO_2_ per hectare of forest land and year. The system with PRI consumes the assimilative capacity of 1.67 ha of forest land in terms of CO_2_, while CMI consume 1.86 ha. To illustrate the footprint area in terms of football pitches, the PRI need 2.4 football pitches to assimilate the CO_2_, while the CMI require 2.7.

The total cost of the system with CMI was SEK 1.3 million. In round figures, the GDP in Sweden in 2006 was SEK 3000 billion. The total emission of CO_2_ equivalents through final energy use was 63 billion kg^3^ (Hellstrand 2015). The emissions per SEK in the Swedish economy were therefore 21 g of CO_2_ equivalents. This suggests that the indirect emissions of CO_2_ for the alternative of CMI were 27,300 kg, i.e. twice the amount from the direct emissions. This indicates that when estimating the ecological dependence of economic systems, not the least systems that are substantial in economic terms, there is a need to consider both the direct support and the indirect support from nature.

In Table 3, a route of calculation is used by which the amount of emissions from different substances from energy use can be estimated. These emissions affect most of the 16 Swedish national environmental quality objectives (Miljöportalen 2016) and a number of the 17 SDGs of the UN (United Nations 2015).

One way to link emissions from a system to the affected ecosystems is presented above. This exemplifies a general route by which the appropriation by any economic system of ecosystem capacity to deliver any kind of natural resource and any kind of waste-assimilative capacity can be quantified. The toolbox for sustainable development presented in Hellstrand (Hellstrand 2015) provides instruments for doing this. Further research should focus on how to obtain data on how people travel to and from healthcare.

Covid-19 stresses the importance of (1) a secure supply of insoles and shoes and (2) the need to minimise travelling and social interaction (Rogers et al. 2020). For PRI, (1) is a disadvantage and (2) an advantage.

The study has limitations. First, the estimate of the costs of treating DFUs relates to 1990 (Apelqvist et al. 1995). In spite of this, it is the most accurate information, to our knowledge. This underlines the importance of updating the work by Apelqvist et al. with accurate information. Second, in the effective prevention of DFUs, the supply of insoles should go hand-in-hand with interventions such as access to podiatry, information on self-care, foot checks and access to multidisciplinary services (International Working Group on the Diabetic Foot 2019). When calculating the total cost of sustainable prevention, these costs should be considered. Third, one area to study further is the function for patients of insoles produced in different ways, such as CMI, PRI, 3D-printed and semi-custom-made insoles (Healy et al. 2013, 2018; Van Netten et al. 2020).

Finally, we suggest that better information is needed regarding the actual distance patients travel to and from the DPO, the type of transport system and the time of the patient and her/his employer that is consumed.

### Major findings/contributions

1. We adapt to healthcare a general methodology for the quantification of the sustainability performance of systems from single product/service to national level, which is internally consistent and harmonises with the known properties of affected systems. This methodology identifies the contribution to a set of sub-objectives within the sustainability context from low to high system level, within the ecological, economic and social dimensions. It facilitates an analysis of the impact of measures in the healthcare system on national environmental objectives, as well as the global 17 sustainable development goals of the UN. We apply it to the example of supplying insoles to patients with diabetes. The figures that are used are realistic.
2. The study shows the importance of considering the value of the time of the patient and by extension the time of her/his employer that is consumed when designing efficient healthcare systems. In our example, this contributed around one third of the total costs.
3. The results indicate high potential for net savings in the healthcare system where the studied measure of providing insoles to patients with diabetes with this need moves from meeting 5% of this need to 100%, which would result in net savings of 30 (the CMI system) alternatively 40 million SEK (the PRI system) in a region with a population of 500,000 people. The cost of this would be around 25 million SEK in the CMI system and 15 million SEK in the PRI system.
4. The results show the importance of the way the work is organised at low system level in the healthcare sector where one system (PRI) per treatment can reduce the ecological, economic and social footprint by 50%, 46% and 46%, respectively. (The reduction in economic and social footprints is equal, as we adopt a simple welfare-economic analysis, where the economic cost includes impacts on the environment, the healthcare provider and the patients, meaning that this broader economic value is also a measurement of the social value.)

Our major findings are:

- Efficiency in healthcare, in traditional terms, contributes to efficiency in a broader sustainability context
- There is substantial potential for improvements at low system levels that make a difference to sustainable development at high system levels
- In the case studied, the efficiency was almost doubled by reducing patient visits by 50%, while the treatment effects were similar
- Measures substantially improving efficiency at low system levels significantly increase the number of treatments delivered within the given budget restrictions
- With regard to needs, the current supply of insoles to patients with diabetes is far too low, producing costs to patients and to society through an increased level of DFUs
- When designing efficient healthcare systems, the cost of the consumption of patients’ time should be considered
- In the case of the delivery of insoles to patients with diabetes, the results obtained indicate that
  - The environmental impact is of minor importance
  - When measuring the environmental impact, both direct and indirect support from nature, from ecosystems, should be considered
- With a global economy trespassing on ecological sustainability limits and the substantial health costs due to a degraded environment, the demand for methods evaluating links between ecological, economic and social systems is important and it is increasing. The capacity to handle interrelations with human health and healthcare systems is critical.

## Conclusions

By using PRI, compared to CMI, one visit to the healthcare supplier was needed instead of two. The cost/treatment for the healthcare supplier was reduced by 46%, allowing the treatment of 86% more patients within the same budget.

In our example, the costs for the healthcare supplier in the PRI-case was 63% of the total costs, the costs of the consumption of the time of the patients was 33%. The result stresses the importance of considering the costs associated with the time consumed by the patient when optimising healthcare systems.

The paper demonstrates a method with the capacity to quantify impacts on the 17 SDG constituting the 2030 Agenda for Sustainable Development.

## Data Availability

For detailed information on routes of calculation, please contact the corresponding author.

## Abbreviations

DFU: Diabetic foot ulcers
CO_2_: Carbon dioxide
CMI: Custom-made insoles
CPO: Certified prosthetist and orthotist
DPO: Department of Prosthetics and Orthotics
ha: Hectare
NCD: Non-communicable diseases
PRI: Prefabricated insoles
SDG: Sustainable Development Goals
UN: United Nations
WHO: World Health Organisation

## Footnotes

1 Based on UNstat (2020), https://unstats.un.org/unsd/snaama/Downloads, accessed 2020-06-12; in 1990 1 US$ corresponded to 592 SEK.

2 Total emission of carbon dioxide = number or insoles * summary of CO_2_ equivalence (kg).

3 Emissions from biofuels included.
